# Effects and Components of Placebos with a Psychological Treatment Rationale – Three Randomized-Controlled Studies

**DOI:** 10.1038/s41598-018-37945-1

**Published:** 2019-02-05

**Authors:** Jens Gaab, Joe Kossowsky, Ulrike Ehlert, Cosima Locher

**Affiliations:** 1 Division of Clinical Psychology and Psychotherapy, Department of Psychology, University of Basel, Basel, Switzerland; 2 Department of Anesthesiology, Critical Care, and Pain Medicine, Boston Children’s Hospital, Harvard Medical School, Boston, USA; 3 Division of Clinical Psychology and Psychotherapy, Institute of Psychology, University of Zürich, Zürich, Switzerland

## Abstract

In recent years, placebos have evolved from a mean to control for ‘therapeutic chaff’ to something that has clinically relevant effects with biological underpinning and that is considered to have clinical as well as scientific potential. However, the wealth of scientific placebo research is conceptualized in a biomedical context, i.e. based on placebos provided with a biomedical treatment rationale, whereas little is known about effects and mechanisms of placebos provided with a psychological treatment rationale. This has important repercussions not only on placebo research, but also on attempts to establish specificity of psychological interventions, such as psychotherapy. Therefore, we set out to assess the effects and possible components of placebos provided with a psychological treatment rationale in three experiments on healthy subjects. We show that placebos provided with a psychological treatment rationale are effective in short- as well as mid-term, but only when provided by a trustworthy, friendly and empathetic experimenter. These findings indicate that placebos are effective outside the medical context and thus need be controlled for in non-medical trials. Furthermore, it highlights and confirms the importance of a plausible psychological treatment rationale in the context of a therapeutic alliance for psychological interventions, such as psychotherapy.

## Introduction

Placebos are valuable for clinical science in at least two ways. First, they help to scrutinize specific effects of medical interventions in the so-called gold standard of clinical investigation, i.e. randomized placebo-controlled trials^1^. Next, genuine placebo research helps to elucidate placebos’ true effects and mechanisms of treatment components caused by anything other than the active verum, which in turn can be clinically harnessed^2,3^.

In general, placebos are thought to mimic their active counterparts up to the point of indistinguishability, thus controlling for anything but the purposely active ingredient. But whereas placebos in the context of medical interventions, i.e. the classical ‘sugar pill’ provided with a biomedical treatment rationale “This is a potent pain killer, which will reduce inflammation and thus pain”, are extensively tried and tested, placebos provided with a psychological treatment rationale, such as “Working through your conflict will reduce your pain”, have received considerably less empirical scrutiny. Of course, so-called ‘placebo control conditions’, ‘attention controls’, ‘active controls’ or ‘non-directive controls’ are commonly used in trials testing psychological interventions, but have been found to be ripe with conceptual as well as pragmatic problems^4^. Accordingly, this led to varying estimates of specificity relative to the operationalization of the placebo condition and researchers’ allegiance^1,5–8^. Placebo conditions in trials of psychological interventions are not only structurally different and thus clearly distinguishable from its assumed verum comparator, but they often also lack components which are clearly not specific to psychological interventions, such as talking about emotional problems. For example, in a clinical trial of cognitive behavior therapy for depressed elderly patients, therapists in the control condition were instructed to discuss ‘neutral topics such as hobbies, sports, and current affairs. (…)’ with ‘little focus on emotional issues.’ (cited from^9^). Accordingly, the validity of placebos in clinical research on psychological interventions has been questioned^10^ as the main prerequisite for placebo-controlled trials – single or preferable double blinding – is not applicable, ridiculing the goal of the attempt.

Importantly, it needs to be noted that placebo control conditions in psychotherapy trials – even when disregarding their lack of validity – would throw only little light on the effects of placebos provided with a psychological treatment rationale, because their consequences are best considered as placebo responses. The latter are understood to encompass both placebo effects as well as spontaneous remission, regression to the mean, detection ambiguity and unidentified co-interventions, whereas placebo effects are understood as differences between placebo administration and no treatment^11^. Also, the quest for valid placebos provided with a psychological treatment rationale should not be mistaken with placebos provided with a biomedical treatment rationale having effects on psychological parameters, such as the anxiety, disgust, empathy, stress, emotional well-being or social trust^12–19,20^ nor with mindset-changing information having effects on subjective outcome, such as perceived exercise^21^.

However, the comparative lack of research on placebos provided with a psychological treatment rationale is neither warranted nor inconsequential as psychological interventions are often first-line treatments or integral components in the clinical management of mental disorders and considering that their specificity is subject to debate^22^. Thus, it seems non-acceptable from a scientific, clinical, methodological and ethical point of view to abandon attempts to examine effects of placebos provided with a psychological treatment rationale and consequently to estimate specific and unspecific effects in the realm of psychological interventions^23^. Based on these considerations, we set out to assess the effects of otherwise inert interventions provided with a psychological treatment rationale in three independent studies. Furthermore, we tested the modulation of possible placebo effects by the interpersonal behavior of the experimenter. These results will inform the debate on placebos provided with a psychological treatment rationale and scientific attempts to establish specificity of psychological interventions.

## Methods

### Participants and design

Three independent and consecutive randomized controlled experiments in healthy participants were conducted, i.e. the green dot-, green flux- and green morph-experiment. Interested subjects were enrolled amongst students of the University of Zürich (green dot-experiment) and the University of Basel (green flux- and green morph-experiment). Inclusion criteria were being between 18 and 50 years of age, no presence of a mental disorder and no psychological or psychiatric treatment (current or during the last 6 months) as well as no current usage of medication by self-report. Before randomization, interested subjects were informed that the study examines the influence of visual information, but they were not informed that the study encompasses an intervention and its evaluation. Up to randomization, experimenters were naïve about group assignment of the respective participant. To control for any influence of verbal or interpersonal behavior of the experimenters before randomization, verbal and interpersonal behavior of experimenters was manualized (see Appendix) as well as minimal and neutral.

In the green dot-experiment, participants signed the informed consent and completed a baseline assessment of the primary outcome and then were randomly assigned to three experimental conditions (for description see below). After randomization, participants received the intervention placebo according to group assignment. Participants then completed a questionnaire to assess the secondary outcome and underwent the placebo (see below). After the placebo, participants completed the post assessment of the primary outcome and then were debriefed about the aims of the study.

The green flux- and the green morph-experiment consisted of two lab sessions and three assessments of the primary outcome (baseline, post and follow-up). The primary outcome was assessed before the randomization (baseline assessment), before the second session (post assessment) and seven days after the second session (follow-up assessment). At baseline, participants signed the informed consent, completed a baseline assessment of the primary outcome and then were randomly assigned to three experimental conditions (for description see below). Participants then completed a questionnaire to assess the secondary outcome (see below) and underwent the placebo intervention (see below). After the placebo, participants were encouraged to use the placebo as often as possible on an individualized website (see Appendix). Adherence to comply with this instruction as well as individual access to the website was not assessed. After an interval of a median of 3 days (range 2-3 days), participants completed the second assessment of the primary outcome and then underwent the placebo intervention a second time in the lab. After the second session, the placebo intervention was still available on websites (see Appendix), but participants were not further encouraged to use the placebo.

For all experiments, individual assignment was concealed in sequentially numbered envelopes and opened in the presence of participants. Participants were stratified according to sex to control for unequal numbers of male and female participants. Participants who enrolled in the study receive credit points accountable for their Bachelor studies or participated in a draw for cinema tickets. To control for possible experimenter effects, allocation of experimenter to condition was stratified. For all experiments, ethical approval was obtained from the Ethics Committee of the Canton Stadt Basel and Baselland (EKBB; green dot and green flux studies) as well as its successor Ethics Committee of Northwest and Central Switzerland (EKNZ, green morph study) and all three experiments were performed in accordance with the relevant guidelines and regulations. Informed consent was obtained from all participants.

### Experimental conditions

In all experiments and all conditions, all subjects received placebos, which consisted of animated videos. The placebo in the green-dot-experiment consisted of a 5-minute video (http://youtu.be/e_SmspvY6qE) of a moving green circle (4.2 cm diameter, green color: RGB 102, 177, 50), presented full screen on 15-inch Apple MacBook monitor with full screen brightness. The green circle moved in an unpredictable manner, thus making various turns and changes in speed. In the green flux-experiment, the placebo consisted of a 7-minute video (https://www.youtube.com/watch?v=bPt-m15xAEY endlessly looped for 7 minutes) of a moving combination of the colors green, white and yellow, which was shown on the full screen of a 23-inch Apple iMac monitor with full screen brightness. The placebo in the green morph-experiment was 6.49-minute video of a slowly pulsing green and yellow circles in a changing green background (https://www.youtube.com/watch?v=8qyt5k83XHs), which was shown on the full screen of a 23-inch Apple iMac monitor with full screen brightness. None of the placebo videos contained any sound. The placebos were provided either without or with a psychological rational and without or with a trustworthy, friendly and empathetic relationship. Thus, experimental conditions were fashioned in order to manipulate these known components of placebo^24^. In all experiments, subjects were left alone in the laboratory room to watch the respective videos. Adherence to watching the videos was not monitored.

In all three experiments, participants in the ‘control condition’ were told that they are in the control condition and that they had to watch an animated video in order to match the timing of the other groups, which would receive a real intervention. However, the intervention, i.e. placebo, within each experiment was the same in all conditions, but in the ‘control condition’, the placebo was not provided with a psychological treatment rational, i.e. was not introduced as an intervention.

In the green dot- and the green flux-experiment, the two ‘placebo conditions’ differed with regard to behavior of the experimenter only. Thus, participants in both placebo conditions were provided with a psychological treatment rationale on the effects and the scientific evidence for this intervention, including that the beneficial effects of the intervention were not due to biological effects of colors on the brain, but due to psychological processes only, such as the “activation of early conditioned emotional schemata through the color green” (see Appendix). The psychological treatment rationale for the placebo conditions was fashioned to provide a psychological explanation and thus not to evoke any biological or neuroscientific assumptions. Also, while the employed psychological treatment rationale was intended to maximize response expectancy, it was not informed by any real scientific finding or model and all provided “scientific evidence” was made up. However, in contrast to the ‘placebo only’ condition, in which experimenters were instructed to behave neutral and ‘technical’, experimenters in the ‘placebo plus’ condition were instructed to be trustworthy, friendly and empathetic throughout the course of the experiment. This trustworthy, friendly and empathetic interaction of experimenter with participants was restricted to experiments as there was no interaction between experimenters and participant outside the laboratory sessions. All verbal and interpersonal behavior of experimenters in all studies and conditions was manualized (see Appendix).

In contrast to the green dot- and the green flux-experiment, a ‘control plus’ condition was added in the green morph-experiment, in which the placebo was not provided with a psychological treatment rationale, but experimenters were instructed to be trustworthy, friendly and empathetic. The intention to include this ‘control plus’ condition was to test whether possible effects in the ‘placebo plus’ condition were merely caused by the trustworthy, friendly and empathetic behavior of the experimenter.

### Outcome measures

The primary outcome in the green dot-experiment was baseline to post assessment changes in momentary mood, assessed with the Mehrdimensionale Befindlichkeitsfragebogens (MDBF^25^), which is explicitly designed to assess momentary changes with three subscales, i.e. ‘good/bad’, ‘alert/fired’, ‘calm/agitated’ mood. To control for possible sequence effects, parallel versions of the MDBF were randomly allocated in different sequence (A-B vs. B-A). The parallel version of the MDBF contains 12 adjectives of momentary mood (e.g. “good mood”, “relaxed”) with a 1 to 5 scoring.

In the green flux- and the green morph-experiment, primary outcomes were changes in perceived stress, assessed with the German version of the Perceived Stress Scale^26^. The PSS assesses the degree of perceived stressful situations experienced during the preceding days and 14 items are designed to assess how predictable, uncontrollable, and overloaded participants evaluate their lives as being. To be able to assess medium-term effects, we changed the wording of the items to assess how participants perceived their stress in the two preceding days.

Secondary outcome in all experiments was perceived interpersonal behavior of the experimenter and response expectancy to the intervention, assessed with two questions each (see appendix). Since the interaction between experimenter and participants in the control groups in the green dot- and green flux-experiments was minimal and the placebos were not introduced as an intervention, secondary outcome was not assessed in the control conditions in these experiments. However, in the green morph-experiment, secondary outcome was also assessed in the ‘control plus’ condition in order to assess the impact of the experimental manipulation of the experimenters’ interpersonal behavior. Secondary outcome was completed after the manualized instructions and before the placebo administration in the green dot-experiment and after the placebo administration at the second lab session in the green flux- and green morph-experiment.

### Statistics

Group differences in demographic and baseline data were calculated with chi-square or univariate analyses. To examine whether the placebo was inert when administered without a therapeutic rationale or a trustworthy, friendly and empathetic experimenter, time effects in the control group in all experiments were calculated with univariate analyses for repeated measures. Group differences in change scores from baseline were analyzed with multivariate analyses. In case of significance, subsequent univariate analyses for short-term (baseline to post assessment) as well as mid-term (baseline to follow-up assessment) changes were conducted. Least significant difference post hoc tests were used to determine differences between conditions. Secondary outcome was analyzed with univariate analyses. Effect sizes for individual group change scores in the primary outcome were calculated as standardized within group changes and expressed in Cohen’s d ((mean baseline – mean post/follow-up)/pooled standard deviation). Analyses were performed according to the principle of intention to treat, with last observation after the intervention carried forward when follow-up data were missing.

## Results

Overall, N = 421 healthy subjects participated in the three experiments (green dot-experiment N = 85, green flux-experiment N = 78, green morph-experiment N = 258). There was no dropout in the green dot-experiment, but in the green flux-experiment three participants dropped out due to minor illnesses between baseline and post assessment and in the green morph-experiment 19 participants did not complete either the post- (N = 2) or the follow up- assessment (N = 17). Randomization resulted in equally distributed groups (‘control’/‘placebo only’/‘placebo plus’ groups: green dot-experiment N = 28/29/28, green flux-experiment N = 25/25/29; ‘control’/‘control plus’/‘placebo only’/‘placebo plus’ groups: green morph-experiment N = 63/65/65/65), which did not differ significantly (all p > 0.05) in mean age in years (green dot-experiment: 24.6, range 18–44, green flux-experiment 24.6, range 18–45, green morph-experiment 24.01, range 18–49) and sex distribution (female/male: green dot-experiment 82%/18%, green flux-experiment: 79%/21%/, green morph-experiment: 75%/25%). Experimenters were 15 female and one male psychology master students (green dot-experiment 5/0, green flux-experiment: 3/0, green morph-experiment 7/1; see Acknowledgement) and all experimenters had an equal share of participants in all experimental condition (all experiments p > 0.9).

### Effects of condition on primary outcome

**Figure 1:**
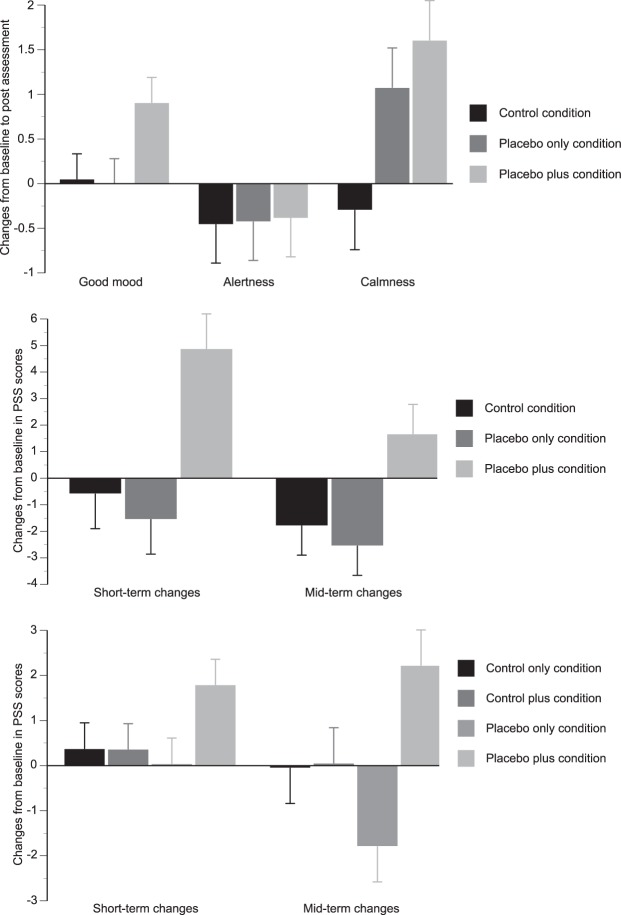
Change scores in MDBF subscale and short- and medium-term change scores PSS score between groups in the green dot- (top), green flux- (middle) and green morph-experiment (bottom). To allow comparability with the results of the green dot-experiment, differences scores in the green flux- and green morph-experiments were inversed, thus positive differences scores indicate reductions in perceived stress. Bars represent mean values and error bars represent standard error of mean.

In the green dot-experiment, moods levels did not change significantly over time in the ‘control condition’, indicating that the placebo had no significant effect on mood per se (F(1/27) = 0.07, p = 0.79). The experimental manipulation resulted in significant baseline to post-placebo assessment changes in MDBF subscales between groups (F(6/162) = 2.4, p = 0.03, see Fig. 1), with significantly differences for mood (MDBF subscale ‘good/bad’ mood: F(2/82) = 3.27, p = 0.05) and calmness (MDBF subscale ‘calm/agitated’ mood F(2/82) = 5.1, p = 0.0.01), but not for alertness (MDBF subscale ‘alert/tired’: F(2/82) = 0.007, p = 0.99). Post hoc tests indicated significant differences in change in calmness between the placebo conditions and the control condition (all p < 0.03) and in mood changes between the ‘placebo plus’ versus the ‘placebo only’ (p = 0.03) and ‘control’ conditions (p = 0.04), whereas the latter two groups did not differ from each other (p = 0.89). Effect sizes between the ‘control’, ‘placebo only’ and ‘placebo plus’ conditions for changes in calmness were d = 0.10, d = 0.68 and d = 0.91 as well as d = 0.03, d < 0.001 and d = 0.68 for mood changes.

In the green flux-experiment, perceived stress levels did not change significantly over time in the ‘control condition’, indicating that the placebo had no significant effect on the primary outcome when not provided with a rationale (F(2/23) = 2.2, p = 0.14). Groups significantly differed in their overall changes in PSS scores (F(4/150) = 5.2, p = 0.001, see Fig. 1), with significant short-term, i.e. baseline to post assessment, changes (F(2/75) = 11.2, p < 0.001) as well as significant mid-term, i.e. baseline to follow-up assessment, changes (F2/75) = 3.1, p = 0.05). Post hoc tests indicated significant differences in short- as well as mid-term changes between the ‘placebo plus’ and both the ‘control’ and the ‘placebo only’ group (all p < 0.05). Effect sizes of short-term changes in PSS scores were d = −0.22, d = −0.19 and d = 1.19 as well as d = −0.39, d = −0.29 and d = 0.27 for long-term changes in the ‘control’, ‘placebo only’ and ‘placebo plus’ conditions, respectively.

In the green morph-experiment, PSS scores did not change significantly over time in the ‘control condition’, indicating that the placebo had no significant effect on the primary outcome per se (F(2/61) = 0.29, p = 0.75). Groups differed significantly in changes in PSS scores from baseline (F(6/504) = 2.4, p = 0.03, see Fig. 1), with no significant differences in short-term changes (F(3/254( = 1.8, p = 0.14), but significant differences in mid-term changes, i.e. from baseline to follow-up assessment, (F(3/254) = 4.6, p = 0.004). For the latter, posthoc analyses indicated significant differences between the ‘placebo plus’ and all other conditions (all p < 0.05), while the latter did not differ from each other (all p > 0.10). Effect sizes of mid-term changes in PSS scores were d = −0.01, d = −0.01, d = −0.23 and d = 0.40 in the ‘control’, ‘control plus’, ‘placebo only’ and ‘placebo plus’ conditions, respectively.

### Effects of condition on secondary outcome

To examine whether the experimental manipulation led to designated effects on perceived interpersonal behavior of the therapist, i.e. in terms of trustworthiness, friendliness and empathy, and response expectancy, mean values in the assessed two questions were examined between conditions in all experiments. In the green dot-experiment, results showed significantly higher perceived interpersonal behavior of the therapist values in the ‘placebo plus’ conditions in comparison to the ‘placebo only’ conditions (F(1/54) = 6.4, p = 0.01, see Table 1), while conditions did not differ in response expectancy to the intervention (F(1/54) = 0.005, p = 0.94, see Table 1). In the green flux-experiment, participants in the ‘placebo only’ and the ‘placebo plus’ conditions differed significantly in perceived interpersonal behavior of the therapist values, with higher scores in the ‘placebo plus’ then the ‘placebo only’ condition (F(1/51) = 7.7, p = 0.01, see Table 1), while groups did not differ in response expectancy to the intervention (F(1/51) = 0.01, p = 0.93, see Table 1). In the green morph-experiment, participants differed significantly in perceived interpersonal behavior of the therapist (F(2/191) = 3.2, p = 0.04, see Table 1), with significantly higher values in both ‘plus’ conditions, i.e. with a trustworthy, friendly and empathetic experimenter, in comparison to the ‘placebo only’ condition (‘control plus’ versus ‘placebo only’: p = 0.04; ‘placebo plus’ versus ‘placebo only’: p = 0.03), while conditions with a trustworthy, friendly and empathetic experimenter did not differ in perceived empathy from each other (‘control plus’ versus ‘placebo plus’: p = 0.90). Conditions did not differ in response expectancy to the intervention (F(2/191) = 1.0, p = 0.37; ‘control plus’ versus ‘placebo only’: p = 0.43; ‘placebo plus’ versus ‘placebo only’: p = 0.56; ‘control plus’ versus ‘placebo plus’: p = 0.43, see Table 1).

**Table 1:** Perceived empathy and plausibility in all experiments (mean (standard error of mean)).

Experiment	Condition	Perceived Empathy	Perceived Plausibility
Green dot	Placebo only	8.8 (0.2)	6.8 (0.4)
	Placebo plus	9.5 (0.2)	6.8 (0.4)
Green flux	Placebo only	8.8 (0.2)	6.8 (0.3
	Placebo plus	9.5 (0.2)	6.7 (0.4)
Green morph	Control plus	6.8 (0.5)	5.3 (0.5)
	Placebo only	6.0 (0.5)	5.6 (0.5)
	Placebo plus	6.9 (0.5)	5.5 (0.5)

## Discussion

We set out to assess the effects of otherwise inert placebo interventions provided with or without a psychological treatment rationale and with a neutral or trustworthy, friendly and empathetic behavior of the experimenter in three consecutive and independent experiments. In all three experiments, the employed placebos were inert when administered without a treatment rationale, but exerted significant effects on primary outcome when provided with a psychological treatment rationale and a trustworthy, friendly and empathetic behavior of the experimenter. Although only tested in the green morph-experiment, the provision of trustworthy, friendly and empathetic relationship alone had no effects on primary outcome per se, indicating that in the respective experiment, the observed placebo effects on primary outcome was not due to the interpersonal behavior of the experimenter. These observed placebo effects differed in size, with large immediate and short-term effects and small to medium mid-term effects. Interestingly, considering that participants in the respective experiments received the same placebo, the provision of a psychological treatment rationale alone, i.e. without a trustworthy, friendly and empathetic interpersonal behavior of the experimenter, as well as the provision of a trustworthy, friendly and empathetic interpersonal behavior of the experimenter alone, i.e. without a psychological rationale, rendered the placebo effectless. Importantly, the experimental manipulation of both, i.e. providing a psychological treatment rationale as well as the interpersonal behavior of the experimenter, led on the one hand to the expected effects and differences in perceived empathy of the experimenter, while on the other hand perceived response expectancy appeared unaffected.

From an empirical perspective, it needs to be noted that the observed placebo effects are of the same magnitude to those seen in comparable populations with similar outcomes, but obtained with placebos administered within a biomedical context, i.e. with a biomedical treatment rationale. For example, Darragh *et al*.^27^ reported that the administration of placebo “antistress’ intranasal serotonin or oxytocin spray” in healthy volunteers resulted in significant reductions in perceived stress, with effect sizes similar to those reported in the green flux- and green morph-experiment, i.e. between d = 0.40 and d = 1.0. Also, Koban *et al*.^14^ reported a reduction in social pain of d = 1.04 after the administration of an otherwise inert nasal spray with biomedical treatment rationale, i.e. that the nasal spray is a “powerful analgesic that is also effective in reducing emotional pain and negative affect” in subjects who experienced a recent unwanted breakup of their romantic relationship. Furthermore, it needs to be noted that the observed placebo effects also show resemblance to those seen in assumingly non-placebo psychological interventions. For example, psychological stress management trainings in healthy populations with comparable designs have been shown to yield reductions in perceives stress of the same magnitude than those obtained in the green flux- and the green morph-experiments^28,29^. Furthermore, although a direct comparison with effect sizes seen in psychotherapy trials is not warranted due to the fact that they are usually obtained in clinical populations, it needs to be noted that the reported effect sizes would also be not unexpected for psychotherapy trials^30^. Thus, our results show for the first time that placebos administered within a psychotherapeutic-like context and with a psychological treatment rationale have effects with approximately the same size as placebos provided within a biomedical context and with a biomedical treatment rationale as well as – assumingly non-placebo – psychological interventions.

From a theoretical perspective, the observation that the employed placebos only had effects when provided with a treatment rationale and in the context of a trustworthy, friendly and empathetic behavior of the experimenter both confirms and expands the current understanding of placebos and also sheds light on psychotherapy. First, studies examining the modulation of treatment effects by the patient-practitioner relationship have a long history. For example, Egbert *et al*.^31^ were able to demonstrate that “encouragement and education” led to a significant reduction in postoperative administration of narcotics, blindly rated objective and subjective report as well as shortened post-operative hospitalization. Likewise, a comparatively early study in primary care patients with minor illnesses showed that a positive consultation led to significantly better outcome in comparison to a negative consultation, irrespective of providing placebo or no treatment^32^. In their hallmark study, Kaptchuk *et al*.^24^ examined the effects of placebo acupuncture in irritable bowel syndrome, showing that this placebo procedure had moderate effects, which were almost doubled when provided with a friendly and empathetic patient-practitioner relationship. In psychotherapy, the relationship between patients/clients and therapists is an important determinant of outcome^30^, showing its effect irrespective of its perceived importance by the researcher^33^. Also, the effects of an empathetic and supportive relationship have been found to be clinically relevant, equaling the effects of bona fide psychotherapies after controlling for researchers’ allegiance^6^. On the basis of the results of the green morph-experiment, we reason that neither a therapeutic treatment rationale nor the provision of trustworthy, friendly and empathetic relationship alone is sufficient to obtain an effect nor that they are additive, but that these two intervention components interact synergistically. It is important to keep in mind that in interventions the therapeutic relationship is not aimless, but always embedded in a therapeutic setting, i.e. provided with a therapeutic rationale. Considering the importance of the therapeutic alliance for psychotherapy, it is interesting to note that – in contrast to placebos provided with a biomedical treatment rationale^24^ – placebos with a psychological treatment rationale do not show any effect when provided in a neutral and technical way. Thus, it seems that the alliance becomes more important when the rationale is provided within a psychotherapeutic-like context. This finding resembles the results of a recent placebo study demonstrating that the effects of expectations – on the basis of a biomedical treatment rationale – are moderated by warmth and competence of the care provider^34^. However, while the results of the green morph-experiment support the importance of a trustworthy, friendly and empathetic relationship, we did not observe any effects of a trustworthy, friendly and empathetic relationship on response expectancies, indicating that these factors are independent from each other. It is possible that these differences between studies employing placebos with either a biomedical or psychological treatment rationale are due to differences in their familiarity, i.e. that taking the proverbial (placebo) pill is different from participating in a (placebo) psychological treatment in terms of having previous experiences and also possible with regard to the extent to which the respective intervention is socially grounded.

From a methodological point of view, the call to abandon randomized placebo-controlled trials in psychotherapy research is well founded^10^, but that does not imply that the pursuit for specificity is futile. Rather, the examination of placebos provided with psychological treatment rationales would not only offer insights into the potential and the limits of expectancy and plausibility in the context of a therapeutic alliance, but also help to examine effects and mechanisms of these treatment components, regardless of their definition as being characteristic or incidental to a given treatment theory^35^. Considering that the existence and size of specific effects of psychological interventions when compared to placebo conditions is functional to the operationalization of the latter^5,36^, still too little is known about the effects and mechanisms of placebos provided with a psychological treatment rationale.

Several aspects need critical examination. First, it can be argued that neither the intervention nor the way the relationship between experimenter and participant, resembles psychotherapy or the relationship between patients/clients and therapists in psychotherapy. However, with regard to the former, psychotherapy can take many forms, and as such, it is not defined by methods, but rather by its main goal to change feelings and behaviors. In this line of reasoning, our placebo interventions could well qualify as possible psychotherapeutic interventions. With regard to the latter, again, there is no universal definition of the relationship between patients/clients and therapist in that psychotherapists and psychotherapies vary in the degree they rely on, position or use the relationship. Also, although our attempts to experimentally manipulate the relationship quality can be seen as pragmatic and superficial, it led to designated effects in the subjective perception of participants and also, it has been shown that “even (…) “superficial psychotherapy” (…) has very powerful effect(s) on patients (with postoperative pain)” (quote from^31^, quotation mark in original, parenthesis by authors). Second, our attempts to standardize the interpersonal behavior of the experimenters were guided by the caution not to over-regulate the interpersonal encounter. Thus, and for example, we did not standardize the frequency or intensity of eye contact or number of questions answered by the experimenters and it is possible that the interpersonal encounter differed in these aspects between participants and also between experimenters. Therefore, although we were able to validate our approach by its effects on the secondary outcome, it is possible that this outcome was caused by different interpersonal behavior and processes. Third, we did not include a ‘control plus’ condition in the green dot- and the green flux-experiment and thus we can only speculate whether the observed effects of the ‘placebo plus’ conditions are due to the interaction of response expectancy and trustworthy, friendly and empathetic interaction of experimenter with participants or solely driven by the later. Fourth, our primary outcome was restricted to subjective parameters, i.e. momentary mood and perceived stress by self-report. Although this kind of assessment and outcome is customary in the evaluation of psychological interventions, the inclusion of physiological parameters would have corroborated our effects on subjective outcome. Fifth, we did not assess the secondary outcome in control conditions as the used items explicitly referred to an interventional context, e.g. *I feel that the therapist is trustworthy*. As both the use of this wording would have changed the provided rational for the control conditions as much as a rephrased wording would have rendered these items incomparable between conditions, we did not assess secondary outcome in the control conditions. However, the verbal and interpersonal behavior of experimenters in the control conditions were manualized as in all other conditions (see Appendix). Finally, we tested only healthy participants to increase internal validity. Although this might preclude direct implications for clinical research, we are confident that our results are informative for both placebo and psychotherapy research and that in future, this should be expanded also to clinical populations. Also, it needs to be noted that we employed three different variations of the same theme, i.e. ‘green’ and ‘moving’. The majority of participants were psychology students at the Universities of Basel and although participants were asked not disclose study details to fellow students, we changed the appearance of the placebos between experiments to minimize possible unblinding effects.

In conclusion, our results show that placebos with a psychological treatment rationale are possible and effective, when provided in a trustworthy, friendly and empathetic relationship, at least in healthy subjects. This opens the door for genuine placebo research in the realm of psychotherapy and the results of this research would not only be of interest for placebo research, but also inform the conceptualization and testing of psychological interventions.

## Supplementary information

Appendix

## References

[1] Jones DS, Podolsky SH (2015). The history and fate of the gold standard. Lancet.

[2] Enck P, Bingel U, Schedlowski M, Rief W (2013). The placebo response in medicine: Minimize, maximize or personalize?. Nature Reviews Drug Discovery.

[3] Wager TD, Atlas LY (2015). The neuroscience of placebo effects: connecting context, learning and health. Nature Reviews of Neuroscience.

[4] Kirsch I (2005). Placebo psychotherapy: Synonym or oxymoron?. Journal of Clinical Psychology.

[5] Baskin TW, Tierney SC, Minami T, Wampold BE (2003). Establishing specificity in psychotherapy: A meta-analysis of structural equivalence of placebo controls. Journal of Consulting and Clinical Psychology.

[6] Cuijpers P (2012). The efficacy of non-directive supportive therapy for adult depression: A meta-analysis. Clinical Psychology Review.

[7] Dragioti E, Dimoliatis I, Evangelou E (2015). Disclosure of researcher allegiance in meta-analyses and randomised controlled trials of psychotherapy: a systematic appraisal. BMJ Open.

[8] Woodworth RJ, O’Brien-Malone A, Diamond MR, Schüz B (2017). Web-Based Positive Psychology Interventions: A Reexamination of Effectiveness. Journal of Clinical Psychology.

[9] Serfaty MA (2009). Clinical effectiveness of individual cognitive behavioral therapy for depressed older people in primary care: A randomized controlled trial. Archives of General Psychiatry.

[10] Kirsch I, Wampold BE, Kelley J (2016). Controlling for the Placebo Effect in Psychotherapy: Noble Quest or Tilting at Windmills?. Psychology of Consciousness: Theory, Research, and Practice.

[11] Benedetti F, Carlino E, Pollo A (2011). How placebos change the patient’s brain. Neuropsychopharmacology.

[12] Darragh M (2016). A take-home placebo treatment can reduce stress, anxiety and symptoms of depression in a non-patient population. Australian and New Zealand Journal of Psychiatry.

[13] Ellingsen D-M (2013). Placebo improves pleasure and pain through opposite modulation of sensory processing. Proceedings of the National Academy of Science USA.

[14] Koban L, Kross E, Woo CW, Ruzic L, Wager TD (2017). Frontal-Brainstem Pathways Mediating Placebo Effects on Social Rejection. Journal of Neuroscience.

[15] Meyer B (2015). Neural mechanisms of placebo anxiolysis. Journal of Neuroscience.

[16] Petrovic P (2005). Placebo in emotional processing—induced expectations of anxiety relief activate a generalized modulatory network. Neuron.

[17] Rütgen M (2015). Placebo analgesia and its opioidergic regulation suggest that empathy for pain is grounded in self pain. Proceedings of the National Academy of Science USA.

[18] Schienle A, Ubel S, Schöngaßner F, Ille R, Scharmüller W (2009). Disgust regulation via placebo: an fMRI study. Social Cognitive and Affective Neuroscience.

[19] Yan X, Yong X, Huang W, Ma Y (2018). Placebo treatment facilitates social trust and approach behavior. Proceedings of the National Academy of Science USA.

[20] Koban L, Jepma M, Geuter S, Wager TD (2017). What’s in a word? How instructions, suggestions, and social information change pain and emotion. Neuroscience & Biobehavioral Reviews.

[21] Crum AJ, Langer EJ (2007). Mind-set matters: exercise and the placebo effect. Psychological Science.

[22] Goldfried MR (2013). What should we expect from psychotherapy?. Clinical Psychology Review.

[23] Lilienfeld SO, McKay D, Hollon SD (2018). Why randomised controlled trials of psychological treatments are still essential. Lancet Psychiatry.

[24] Kaptchuk TJ (2008). Components of placebo effect: Randomised controlled trial in patients with irritable bowel syndrome. British Medical Journal.

[25] Steyer R, Schwenkmezger P, Notz P, Eid M (1994). Testtheoretische Analysen des Mehrdimensionalen Befindlichkeitsfragebogens (MDBF). Diagnstica.

[26] Cohen S, Kamarck T, Mermelstein R (1983). A global measure of perceived stress. Journal of Health and Social Behavior.

[27] Darragh M, Booth RJ, Consedine NS (2016). ‘Oxytocin’ for the outwardly oriented: Evidence for interactive effects in placebo responding. Journal of Psychosomatic Research.

[28] Gaab J (2003). Randomized controlled evaluation of the effects of cognitive-behavioral stress management on cortisol responses to acute stress in healthy subjects. Psychoneuroendocrinology.

[29] Storch M, Gaab J, Küttel Y, Stüssi AC, Fend H (2007). Psychoneuroendocrine effects of resource-activating stress management training. Health Psychology.

[30] Wampold, B. E. & Imel, Z. E. *The great psychotherapy debate: Models, methods, and finding*s. New York, NY: Routledge, 2. Edition (2015).

[31] Egbert LD, Battit GE, Welch CE, Bartlett MK (1964). Reduction of postoperative pain by encouragement and instruction of patients. A study of doctor-patient rapport. New England Journal of Medicine.

[32] Thomas KB (1987). General practice consultations: is there any point in being positive?. British Medical Journal.

[33] Flückiger C, DelRey AC, Wampold BE, Symonds D, Horvath AO (2012). How central is the alliance in psychotherapy? A multilevel longitudinal meta-analysis. Journal of Counseling Psychology.

[34] Howe LC, Goyer JP, Crum AJ (2017). Harnessing the Placebo Effect: Exploring the Influence of Physician Characteristics on Placebo Response. Health Psychology.

[35] Grünbaum A (1981). The placebo concept. Behaviour Research and Therapy.

[36] Kazdin AE, Wilcoxon LA (1976). Systematic desensitization and nonspecific treatment effects: A methodological evaluation. Psychological Bulletin.

