# Supplementary material for: Effects and Components of Placebos with a Psychological Treatment Rationale – Three Randomized-Controlled Studies
Source: Sci Rep. 2019 Feb 5;9:1421. doi: 10.1038/s41598-018-37945-1 (PMC6363794; doi:10.1038/s41598-018-37945-1)
Supplement: Supplementary file 1 — Appendix [file 41598_2018_37945_MOESM1_ESM.docx]

**EFFECTS AND COMPONENTS OF PLACEBOS WITH A PSYCHOLOGICAL TREATMENT RATIONALE – THREE RANDOMIZED-CONTROLLED STUDIES**

Jens Gaab, Joe Kossowsky, Ulrike Ehlert, Cosima Locher

**APPENDIX**

Primary outcomes

Manual of verbal and interpersonal experimenter behavior

**Primary outcomes**

**Green dot experiment:** Mehrdimensionale Befindlichkeitsfragebogens (MDBF, Steyer et al., 1997)

*At the moment, I feel content/rested/restless/bad/nerveless/relaxed/tired/good/agitated/awake/unwell/relaxed*

Scoring: 1=*not at all* to 5=*very much*

**Green flux experiment:** Perceived Stress Scale (PSS, 14 item version; Cohen et al., 1983)

*In the last two days, how often have you been upset because of something that happened unexpectedly?*

*In the last two days, how often have you felt that you were unable to control important things in your life?*

*In the last two days, how often have you felt nervous and “stressed”?*

*In the last two days, how often have you dealt successfully with irritating life hassles?*

*In the last two days, how often have you felt that you were effectively coping with important changes that were occurring in your life?*

*In the last two days, how often have you felt confident about your ability to handle your personal problems?*

*In the last two days, how often have you felt that things were going your way?*

*In the last two days, how often have you found that you could not cope with all the things that you had to do?*

*In the last two days, how often have you been able to control irritations in your life?*

*In the last two days, how often have you felt that you were on top of things?*

*In the last two days, how often have you been angered because of things that happened that were outside of your control?*

*In the last two days, how often have you found yourself thinking about things that you have to accomplish?*

*In the last two days, how often have you been able to control the way you spend your time?*

*In the last two days, .how often have you felt difficulties were piling up so high that you could not overcome them?*

Scoring: 0=*never*; 1=*almost never*; 2=*sometimes*; 3=*fairly often*; 4=*very often*

**Secondary outcome: Perceived interpersonal behavior of the experimenter and response expectancy to the intervention**

Scale ‘*Interpersonal behavior of the experimenter’*:

1. *I feel that the therapist is trustworthy*
2. *The therapist is friendly and empathetic*

Scale ‘Response expectancy’:

1. *I think that this intervention can lead to a positive change of my current condition*
2. *I am convinced that I will respond to this intervention*

Scoring: 1=*not at all* to 5=*very much*

**Manuals of verbal and interpersonal** **experimenter behavior**

**Green dot-experiment**

‘control only’ condition

Interpersonal behavior of experimenters: *The experimenter will introduce herself/himself and behaves neutral and in a matter of fact manner towards the participant. The experimenter will not answer questions due to lack of time.*

Content of the verbal instructions (abbreviated): *You are in the control group of a study investigating the effects of psychological factors on perception. As you are in the control group, you only need to watch a video. This video has no known effects on your mood, but it might be possible that you temporarily get a little tired. The participants in the other study groups will receive a real Cromotherapy intervention. However, you are in the control group and your control intervention will not have any effects. You are going to see a film. I will leave you alone watching the film for five minutes and when the film has ended, I will come back to you.*

‘placebo only’ condition

Interpersonal behavior of experimenters: *The experimenter will introduce herself/himself and behaves neutral and in a matter of fact manner towards the participant. All information on the theory behind the intervention and the introduction of the intervention will be read. The experimenter will not answer questions due to lack of time.*

Content of the verbal instructions (abbreviated): Same as in ‘placebo plus’ condition (see below)

‘placebo plus’ condition

Interpersonal behavior of experimenters: *The experimenter is interested to create a positive contact and shows unconditional regard to the participant. The experimenter answers questions with interest and leads through the experiment in a trustworthy, friendly and empathetic* *manner. The participant should feel well and cared for. The participant should be greeted and called by her/his named. At the second session, the experimenter will ask for any possible changes since the first session and acknowledges this with eye contact and smiling.*

Content of the verbal instructions (abbreviated, content is read from paper):

- Introduction: *Thank you for participating in this study. I will briefly introduce myself and the explain the theory behind the intervention as well as describe the intervention itself.*
- Introduction of experimenter: *My name is X.X. and I am happy to be participate in this study on color and mood, with which is also part of my master thesis. As part of this study, I was able to complete a two-week training at the “Colour Psychology Unit” of the University of London. There, I was introduced to EMCIT, which stands for “Eye Movement Colour Impact Therapy”, so that I am now able to perform and also analyze this intervention. To me, this is a very expedient and also effective add-on to other effective psychotherapy interventions. Also, it is also easy to administer.*
- Information about the underlying theory: *Now, I will explain the underlying theory to you. The effects of color have ever since played an important role for humans The central psychological explanation for this is that visual information are incorporated into psychological schemata at a very early stage of development. These schemata are understood as psychological units, which organize our emotions, behaviors and experiences and as such, they are also central starting points for psychotherapy. While this theory has long received little scientific interest, things changed when a group of researchers at Harvard University were able to show that our visual perception in fact has a very strong effect on mood. This then got published in Science, which set off a true research boom and which led to the discovery that the color green truly has a profound beneficial effect on mood, especially in depressive patients. This is explained by the positive symbolization of green in our western society, which is understood to represent calmness as well as good mood. Interestingly, these effects cannot be explained biologically or neurologically as for example the color green does not have the same effects in Inuit, which makes them feel uneasy. This indicates that culture-specific and very early childhood experiences and unconscious processes are at the core of this phenomenon. This specific effect of color has important therapeutic implications, as for example seen in larger psychotherapy effects in green seasons, i.e. spring and summer, and lower depression rates in rural areas, where people are in closer contact to nature. Also, the so-called winter depression, which usually ends as soon as trees start to green in spring, can be explained by this effect. All in all, this all shows that color therapy is a promising approach to improve mood and treat hopelessness. As a consequence, the color green is now used to support treatments for depression in clinical practice. Do you have questions so far? It is important for me that this is comprehensible for you!*
- Information about the intervention: *Now to our therapy! In our study, we are testing the EMCIT, which was developed in cooperation with the* *“Colour Psychology Unit” of the University of London. In this method, which also was tested successfully in pilot studies, we combine the color with movement. As a consequence, the psychological meaning of the color green is augmented and its psychotherapeutic power is fully utilized. This has been successfully demonstrated in depressive populations and also in a study on students in England. We now want to examine this in a large healthy population in Switzerland.*
- Instructions for the intervention: *I want you to follow the green dot with your eyes. Try to concentrate on the color and let the green dot have its fully impact on you. Allow all thoughts and pictures and try to immerse yourself in the green color and the movement. The more you engage, the more you will profit from it. The scientific results so far clearly show that those with a very good visual imagination will benefit from this therapy. Do you have further questions?*

Green flux-experiment

‘control only’ condition: Same as in green dot-experiment

‘placebo only’ condition: Same as in green dot-experiment, except that both the colors green and yellow is introduced as having beneficial effects, as the employed ‘green flux’ video contains the colors green and yellow.

‘placebo plus’ condition: Same as in green dot-experiment, except that both the colors green and yellow is introduced as having beneficial effects, as the employed ‘green flux’ film contains the colors green and yellow.

Green morph-experiment

‘control only’ condition: Same as in green dot- and green flux-experiment

‘control plus condition: Contentwise same as in the ‘control only’ condition but with interpersonal behavior of experimenters as in the ‘plus’ condition

‘placebo only’ condition: Same as in green dot- and green flux-experiment, except that both the colors green and yellow is introduced as having beneficial effects, as the employed ‘green flux’ film contains the colors green and yellow.

‘placebo plus’ condition: Same as in green dot- and green flux-experiment, except that both the colors green and yellow is introduced as having beneficial effects, as the employed ‘green flux’ film contains the colors green and yellow.
